# Toxocarose oculaire: à propos de deux cas et revue de la literature

**DOI:** 10.11604/pamj.2014.17.71.3823

**Published:** 2014-01-30

**Authors:** Chama Daoudi, Mina Laghmari, Kamal Naciri, Hanane Handor, Zouhir Hafidi, Chaimae Hajji, Rajae Daoudi

**Affiliations:** 1 Université Mohammed V Souissi, Service d'Ophtalmologie A de l'Hôpital des Spécialités, Centre Hospitalier Universitaire, Rabat, Maroc

**Keywords:** Toxocarose, Toxocara canis, ELISA, oeil, toxocariasis, Toxocara canis, ELISA, eye

## Abstract

*Toxocara canis* est un nématode de la famille des Ascaridés, il peut être responsable de manifestations oculaires et générales lors d'une contamination accidentelle dans le cadre d'une pathologie du “péril fécal“, les atteintes oculaires sont plus fréquentes chez l'enfant en raison du contact souvent répété avec de jeunes animaux favorisant ainsi la dissemination de cette pathologie dite des “mains sales“, nous rapportons deux cas d'enfants présentant une toxocarose oculaire à granulome postérieur, négatif pour le sérodiagnostic spécifique. La réalisation de la ponction de la chambre antérieure et d'un test ELISA par antigènes homologues de *Toxocara canis* sur l'humeur acqueuse ont permis de poser le diagnostic formel.

## Introduction

La toxocarose oculaire est une parasitose liée à l'infection des tissus oculaires par la larve d'un nématode de la famille des Ascaridés: *Toxocara canis* ou plus rarement *Toxocara catis*, également responsable du syndrome de larva migrans viscéral. Cette pathologie transmise par les jeunes animaux est le plus souvent rencontrée chez les jeunes enfants. Ils existent trois formes cliniques principales: la pan uvéite, le granulome postérieur et le granulome périphérique. Il faut savoir y penser devant tout “foyer choriorétinien blanchâtre à type de granulome“. Le diagnostic de certitude repose sur l’étude immunologique des liquides intraoculaires. Nous rapportons deux cas de toxocarose oculaire à forme de granulome postérieur chez deux enfants séronégatifs pour cette helminthiase.

## Patient et observation

**Figure 1:**
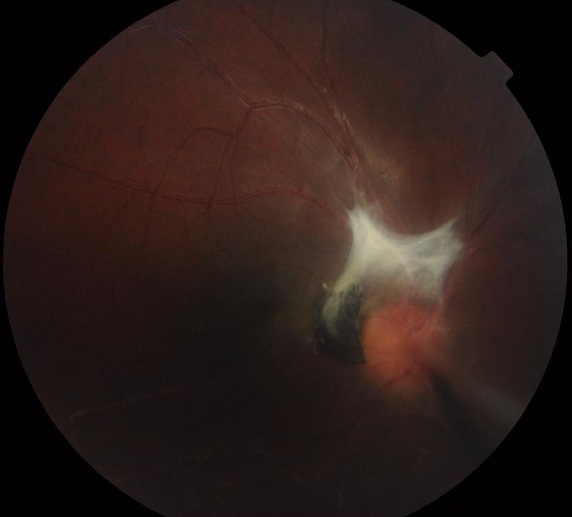
Foyer blanc grisâtre partiellement pigmenté inter papillomaculaire à limites nettes empiétant sur la papille entourée d'un soulèvement rétinien tractionnel avec bride vitréenne dirigée vers la périphérie nasal, macula de siège ectopique

**Cas clinique 1:** Il s'agit d'un enfant âgé de 12 ans, admis pour rougeur oculaire récidivante de l'oeil droit associée à une douleur et photophobie. Dans ses antécédents, il a présenté 4 mois avant son admission un traumatisme par jet de pierre au niveau de l'oeil droit, il a également une notion de contact avec les chiens. L'examen clinique à l'admission objectivait une acuité visuelle à l'oeil droit de mouvement des doigts non améliorable et à l'oeil gauche de 10/10 Parinaud 2. L'examen à la lampe à fente retrouvait à droite une cornée siège d'une keratopathie en bandelette avec précipités retro cornéens, un tyndall + de la chambre antérieure, iris de trame et de coloration normales, des synéchies irido-cristalliniennes à 5h et 8h avec une hyalite +. Le fond de l'oeil retrouvait au pôle postérieur, un foyer blanc grisâtre partiellement pigmenté inter papillomaculaire à limites nettes empiétant sur la papille entouré d'un soulèvement rétinien tractionnel avec bride vitréenne dirigée vers la périphérie nasal, macula de siège ectopique, absence d'autres foyers périphériques (Figure 1). L'examen à la lampe à fente ainsi que le fond de l'oeil ne retrouvaient aucune anomalies de l'oeil gauche. Les bilans biologiques réalisés au service objectivaient une hyperéosinophilie à 850/mm3, la radiographie thoracique était normale et l'intradermoréaction à la tuberculine négative. Les sérologies sanguines étaient négatives à la fois pour la toxoplasmose (IgG et IgM) et pour la toxocarose (*Toxocara canis* Technique ELISA=0,481). Sur le prélèvement d'humeur aqueuse, la recherche par réaction de polymerisation en chaine (PCR) de l'ADN de toxoplasmose était négative, et l'immunodiagnostic de toxocarose par ELISA positive à 1,814 (valeur seuil à 0,478). Le WesternBlot a également confirmé la positivité. On a réalisé également une radiographie des orbites et une tomodensitométrie orbito-cérébrale devant la notion de traumatisme oculaire, qui se sont avérées normales. Un traitement adapté a été instauré comprenant une corticothérapie générale (prédnisone 1mg/kg/j) et un traitement minute antiparasitaire par albendazole. L’évolution a été marquée par une régression rapide de l'uvéite mais le pronostic visuel apparaissait péjoratif pour cet oeil vue la localisation du foyer et l'importance des brides vitréennes.

**Figure 2:**
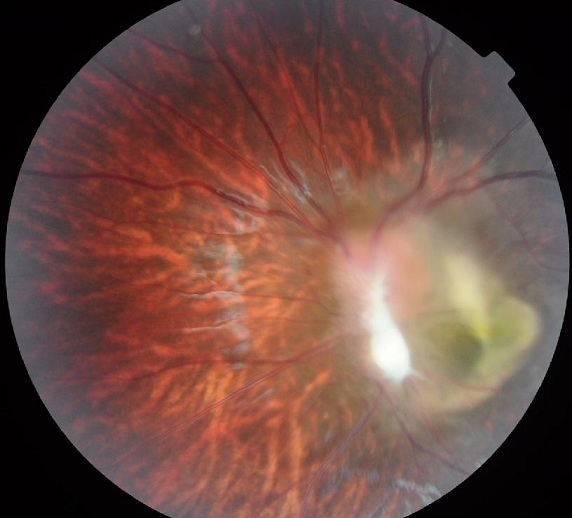
Granulome inflammatoire blanc jaunâtre inter papillomaculaire profond, sous retinien entouré par une zone d'atrophie avec décollement séreux rétinien sus jacent

**Cas clinique 2:** Il s'agit d'un enfant âgé de 4 ans, l'ainé d'une fratrie de 2, admis pour une esotropie de l'oeil gauche, il n'a aucun antécédent particulier ni de cas similaire dans la famille. L'examen clinique à l'admission objectivait une acuité visuelle à l'oeil droit de 10/10, et à l'oeil gauche à mouvement des doigts non améliorable. L'examen à la lampe à fente ainsi que l’étude du fond de l'oeil ne retrouvaient à droite aucune anomalie. L'oeil gauche présentait un strabisme convergent, oeil droit fixateur, cornée claire, bonne chambre antérieure, pas de synéchies. Le fond de l'oeil retrouvait un granulome blanc jaunâtre inter papillomaculaire profond, sous rétinien entouré par une zone d'atrophie avec décollement séreux rétinien sus jacent, on ne note pas de bride ni de foyer actif ou cicatriciel (Figure 2). Les bilans biologiques réalisés au service objectivaient une anémie hypochrome microcytaire, la radiographie thoracique était normale et l'intradermoréaction à la tuberculine négative. Les sérologies sanguines étaient négatives à la fois pour la toxoplasmose (IgG et IgM) et pour la toxocarose (*Toxocara canis* Technique ELISA=0,481). Sur le prélèvement d'humeur aqueuse, la recherche par réaction de polymerisation en chaine (PCR) de l'ADN de toxoplasmose était négative, et l'immunodiagnostic de toxocarose par ELISA positive à 1,482 (valeur seuil à 0,623). Le WesternBlot a également confirmé cette positivité. On a realisé une échographie oculaire qui a montré une petite formation échogène, hétérogène à limites irrégulières formant une saillie papillaire volumineuse sans calcification visible. Un traitement a été instauré à base d'albendazole minute, le traitement antiparasitaire a été réalisé en milieu hospitalier car une réaction anaphylactique due à la lyse parasitaire quelques heures après la prise du traitement est possible.

## Discussion

*Toxocara canis*, nématode parasite du chien, est le plus fréquemment en cause. Il peut s'agir également du *Toxocara cati*, nématode du chat, ou plus rarement, de larves d'animaux sauvages à l'origine de syndromes cliniquement proches [1]. Le cycle du *Toxocara canis* est complexe, différent selon l’âge du chien parasité [2]. Chez le chiot, la contamination peut se faire par l'ingestion d'oeufs embryonnés présents dans les aliment souillés par des excréments parasités entrainant la formation d'adultes matures dans la lumière de l'intestin grêle en 3 à 4 semaines. Les oeufs produits par ces adultes, jusqu’à 200 000 par jour, seront disséminés par les selles des chiots dans le milieu extérieur, où ils deviendront infestants. Chez le chien adulte, l'ingestion d'un oeuf embryonné donne naissance à une larve qui pourra survivre plusieurs mois ou années dans le tissu de l'hôte. L'infestation humaine se fait donc à partir du tube digestif vers la veine porte puis les larves migrent dans les veines pulmonaires et sont disséminées dans les tissus en particulier hépatique, pulmonaire, musculaire, oculaire et nerveux [3]. Des granulomes à éosinophiles se constituent autour de la larve et survient alors une réaction inflammatoire antigénique. L'homme se contamine en absorbant les oeufs embryonnés ou les larves [4], les jeunes enfants se contaminent en portant à la bouche la terre ou le sable souillés par les déjections. La toxocarose oculaire chez l'homme est une maladie reconnue seulement depuis cinquante ans, on doit l'identification de ces nématodes à Wilder en 1950, et c'est en 1960 qu'Ashton décrit le premier cas de granulome rétinien à *Toxocara canis* [4].

Sur le plan clinique, cette parasitose peut se manifester par un granulome du pôle postérieur, un granulome périphérique, une endophtalmie chronique ou une pars planite. Il s'agit le plus souvent d'une uvéite postérieure unilatérale, mais des formes bilatérales ont été décrites [5], avec présence d'un granulome profond du pôle postérieur ou de la périphérie. Chez l'enfant, l'uvéite postérieure se présente volontiers avec un foyer choriorétinien maculaire ou interpapillo-maculaire, tandis que chez l'adulte le foyer est souvent plus périphérique, s'y associent typiquement des tractions vitréo-rétiniennes importantes (comme le montre notre cas), qui peuvent se compliquer de décollement de rétine.

Du point de vue épidémiologique, ce sont surtout les enfants qui sont atteints en raison de leur contact fréquent avec de jeunes animaux et de la pathologie des “mains sales“, elle constitue 1% à 2% des uveites de l'enfant. La moyenne d’âge est de 7.5 ans (entre 2 ans et 31 ans) et 80% ont moins de 16 ans [6]. Le diagnostic repose sur l'anamnèse et la présentation clinique mais doit être confirmé par la présence d'immunoglobulines G spécifiques dans le sérum, l'humeur aqueuse ou le vitré par technique immuno-enzymatique type ELISA, cette dernière offre une sensibilité et spécificité proches des 90% [7]. Il faudrait rappeler qu'il existe des formes séronégatives: sur une série de dix-sept patients, Schantz et al, retrouvent une sérologie négative chez six d'entre eux (35%) [8], ceci s'explique par le fait que la toxocarose oculaire est en général isolée avec une masse antigénique parasitaire emprisonnée dans le granulome oculaire. Ainsi, une sérologie négative n’élimine en rien le diagnostic [9]. Il est donc impératif devant toute suspicion clinique de toxocarose oculaire de rechercher les anticorps spécifiques par ELISA dans l'humeur aqueuse ou le vitré. Rappelons qu'une hyper-éosinophilie sanguine pourrait conforter un doute diagnostique. Les techniques d'imagerie permettent d'aider au diagnostic quand le fond d'oeil est inaccessible, par la recherche de complications telles que le décollement rétinien tractionnel [10], et surtout d’éliminer l'hypothèse d'un rétinoblastome vue la gravité de cette pathologie chez l'enfant.

Le traitement est d'abord préventif en évitant la recontamination, par la pratique d'un déparasitage systématique tri-annuel des chiens et chats familiers, le lavage des mains après contact avec le sol, proscription de tout léchage de l'enfant par l'animal, désinfection de tout lieu souillé par les déjections canines, cuisson suffisante des abats… Le traitement à visée curative sera guidé par l'acuité visuelle, la sévérité de l'inflammation et la nature réversible ou non de l'atteinte oculaire. Généralement les granulomes périphériques silencieux ou avec réaction inflammatoire minime ne nécessitent pas de traitement [11]. Le traitement médical comporte deux volets, l'administration d'un antiparasitaire spécifique en l'occurrence le thiabendazole en raison de sa diffusion intraoculaire, et le recours à une corticothérapie systémique qui permet à forte dose d'atténuer la réaction inflammatoire vitréenne qui est souvent dense. Les attientes oculaire sévère nécessitent une association de traitement corticoïde agressif et d'Albendazol (800mg/j pour les adultes et 400 mg/j pour les enfants) pendant 2 à 4 semaines [12]. Le traitement chirurgical garde certaines indications notamment la vitrectomie par la pars plana en cas de chirurgie du décollement de rétine ou pour pelage de membrane épirétinienne. Dans d'autres cas la vitrectomie est motivée par la persistance de condensations vitréennes pouvant contenir des résidus granulomateux [13].

## Conclusion

La toxocarose oculaire est fréquente dans les pays en voie de développement, néanmoins, son incidence est sous estimée. Le diagnostic est délicat, un sérodiagnostic positif même très spécifique peut être absent. Les prélèvements oculaires sont d'un intérêt majeur pour confirmer le diagnostic suspecté cliniquement, en particulier par technique de Wester Blot semi-quantitatif.
